# Roof Conservatories

**Published:** 1901-08-31

**Authors:** 

**Affiliations:** 37 Great Dover Street, S.E.


### EDITOR'S LETTER-BOX.

[Our Correspondents are reminded that prolixity is a great bar to publication, and that brevity of style and conciseness of statement greatly facilitate early insertion.]

### ROOF CONSERVATORIES.

MESSRS. FRANKENBERG & CO., manufacturers of "Inodorite," 37 Great Dover Street, S.E., write:—"In your issue of the 3rd inst. you deal with 'Roof Conservatories on Town Houses.' We beg to inform you that we lay roofs which can be utilised as gardens or playgrounds, and, with a view to prove their utility, we are prepared through you to lay a roof on our system not exceeding 100 square yards free of cost on any hospital in London that would care to avail themselves of the offer."

